# Xylanolytic metabolism is regulated by coordination of transcription factors XynR and XylR in extremely thermophilic *Caldicellulosiruptorales*

**DOI:** 10.1128/aem.00516-25

**Published:** 2025-06-04

**Authors:** Mohamad J. H. Manesh, James R. Crosby, Tunyaboon Laemthong, Ryan G. Bing, Stefanie H. Chen, Jason Vailionis, Tania N. N. Tanwee, Ying Zhang, Dmitry A. Rodionov, Michael W. W. Adams, Robert M. Kelly

**Affiliations:** 1 Department of Chemical and Biomolecular Engineering, North Carolina State University 6798, Raleigh, North Carolina, USA; 2 Department of Biological Sciences, North Carolina State University 6798, Raleigh, North Carolina, USA; 3 Department of Cell and Molecular Biology, University of Rhode Island 4260, Kingston, Rhode Island, USA; 4 Sanford-Burnham-Prebys Med, Discovery Institute 580550, La Jolla, California, USA; 5 Department of Biochemistry and Molecular Biology, University of Georgia 1355, Athens, Georgia, USA; Kyoto University, Kyoto, Japan

**Keywords:** *Caldicellulosiruptorales*, hemicellulose, transcriptional regulator, fluorescence polarization, Hill coefficient

## Abstract

Global transcription factors (TFs) control metabolic processes in bacteria to efficiently utilize available carbon. The order *Caldicellulosiruptorales* has drawn interest due to the ability of its members to degrade components of lignocellulosic biomass. Regulatory reconstruction of *Anaerocellum (f. Caldicellulosiruptor) bescii* identified two major global transcription factors for xylan utilization, XynR and XylR, and the corresponding putative transcription factor binding sites. Recombinant versions of XynR (LacI family) and XylR (ROK family) were subjected to fluorescence polarization (FP) and biolayer interferometry (BLI) analysis to confirm the predicted binding sites. Four XynR sites and two XylR sites were validated, accounting for 20 of 26 genes regulated by XynR and six of seven genes regulated by XylR. Bioinformatic analysis of the individual genes controlled by the two regulators showed an inter-dependent scheme for xylan conversion; the transport of xylooligosaccharides (XOS) is dependent on XylR, while enzymes responsible for hydrolysis are controlled by both regulators. For xylose catabolism by the xylose isomerase-xylulose kinase pathway, regulation is also split, with XylR controlling xylose isomerase and XynR controlling xylokinase. The XynR/XylR regulator pair within *A. bescii* is conserved in all sequenced species of *Caldicellulosiruptorales*, suggesting similarities in regulating linear xylan conversion. In other xylanolytic thermophiles, XylR homologs control xylan degradation, compared to just 6 out of 26 genes for *A. bescii*. These results show that two separate regulatory schemes (dual repression) are coordinated by *A. bescii* to effectively regulate the hemicellulose inventory and xylan catabolism.

**IMPORTANCE:**

To take full advantage of extreme thermophiles as platform metabolic engineering microorganisms, the tools for genetic manipulation must be further developed, and strategies that exploit a better understanding of metabolic regulation need to be discerned. *Anaerocellum bescii*, the most studied of the extremely thermophilic fermentative anaerobic bacteria that can utilize microcrystalline cellulose, can degrade microcrystalline cellulose and hemicellulose and has been metabolically engineered to convert the resulting sugars to products such as ethanol and acetone. For xylan, in particular, two major global transcription factors (TFs), XynR and XylR, play a role in sugar metabolism, although their predicted regulatory interdependence from bioinformatics analysis has not been elucidated experimentally. Here, fluorescence polarization (FP) and biolayer interferometry (BLI) were used to explore this issue to support metabolic engineering efforts aimed at improving carbohydrate processing to industrial chemicals.

## INTRODUCTION

Protein-DNA interactions in bacteria control numerous processes including gene expression, replication, foreign DNA recognition, and transcriptional regulation (1). For transcriptional regulation, the protein interaction pair consists of a transcription factor protein (TF) and transcription factor DNA binding site (TFBS) that can either prevent (repressor) or recruit (activator) binding of RNA polymerase (2). Metabolic intermediates or small proteins can act as effector molecules for the binding of TFs, which often cause a conformational change allowing for either dissociation or association of the TF to the TFBS. Identifying both the genes that a putative TF might regulate, as well as the small-molecule effector, provides important physiological insights for an organism, especially for metabolic engineering strategies as well as for understanding cellular processes, such as pathogenesis (1, 2).

Bacteria from the order *Caldicellulosiruptorales* (3–7) have received attention for their ability to deconstruct lignocellulose without pretreatment and have been considered consolidated bioprocessing platforms (5). Of the sequenced strains to date, most efforts for developing an industrial host have focused on *Anaerocellum* (f. *Caldicellulosiruptor*) *bescii* as it is genetically tractable (8) and capable of deconstruction of both cellulose and hemicellulose (3). *A. bescii* encodes cellulases within a single genomic region known as the glucan degradation locus (GDL), while its hemicellulolytic inventory is decentralized over multiple loci (9, 10). Recent metabolic and regulatory reconstruction of carbohydrate catabolic networks in *A. bescii* and closely related genomes identified several putative TFs, including the LacI-family XynR and ROK-family XylR regulators, and their cognate binding sites (11). The reconstructed XynR regulon includes the xylan degradation locus (XDL) genes and four other gene loci encoding uptake transporters and catabolic enzymes predicted to be involved in xylan degradation and utilization of xylose and xylooligosaccharides (XOS). XylR is thought to control xylose isomerase (XylA encoded in *xylA*) and a putative XOS/xylose ABC transporter (XynUVW encoded in *xynUVW*) (11, 12). RNA-sequencing data for *A. bescii* grown on various carbon sources confirmed the upregulation of all predicted XynR target genes on xylan, whereas the predicted XylR-controlled genes were highly upregulated on xylose, xylan, and cellulose (11). However, there are few direct experimental studies on *A. bescii* transcription factors, with only the Rex repressor being characterized experimentally (13). This highlights the need to validate *in silico* predictions for operator sites across the genome to further understand how the regulation influences *A. bescii* metabolism and physiology. Furthermore, additional tools for modulating transcription levels for metabolic engineering, such as inducible promoters, are limited in *A. bescii*, with the xylose-inducible xylose isomerase promoter and its putative operator sites being the only reported examples to date (14).

Here, validation of *in silico* predicted binding sites of two *A. bescii* transcription factors, XynR and XylR, to their putative operator sites from the XDL and other xylose/XOS catabolic gene loci was pursued by fluorescence polarization (FP) and biolayer interferometry (BLI) analysis. This information provides a basis for understanding the dual, but coordinated, regulatory strategy of XOS processing by *A. bescii*. Furthermore, the regulatory strategies employed by *Caldicellulosiruptorales* were compared to those employed by other thermophilic, xylanolytic bacteria. Ultimately, understanding how xylanolytic TFs function will inform the integration of novel regulatory strategies for further metabolic engineering efforts with *A. bescii*.

## RESULTS

### Xylan metabolism is regulated by two independent transcription factors in *A. bescii*

**Fig 1:**
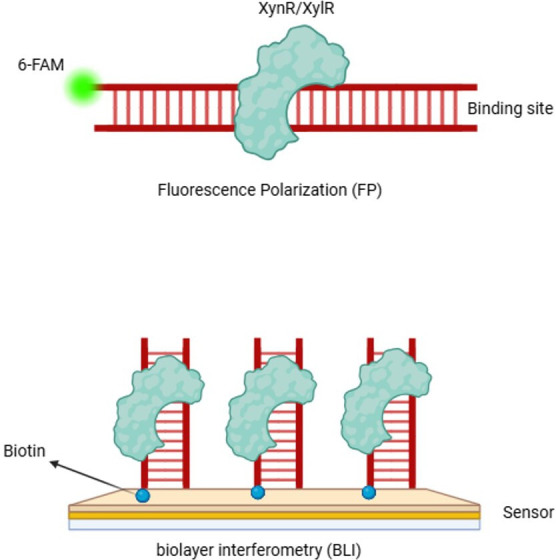
Schematic representation of fluorescence polarization (FP) and biolayer interferometry (BLI) approaches for determining protein-DNA interactions.

To further understand the role of the two putative regulators controlling xylan catabolism in *A. bescii*, full-length versions of XynR (Athe_0566, LacI family) and XylR (Athe_0617, ROK family) were recombinantly produced in *Escherichia coli*. Comparative genomics previously identified putative binding sites for both regulators in the *A. bescii* genome (11) (Table 1). Here, experimental verification was pursued using fluorescence polarization (15) and biolayer interferometry (16) (Fig. 1).

**TABLE 1:** Operator sequences of genes controlled by XynR and XylR

Operator site	Transcription factor	Operator sequence	No. of target genes	Functions
*xynR-xylB*	XynR	aatcgaaatcgcttacaatt	2	Regulator, xylulose kinase
*xyl3A*	XynR	gcttgaaaccgctttctaac	1	β-Xylosidase
*xynMNC*	XynR	tcatgaaatcgctttcctat	3	Endo-xylanase, carbohydrate esterases
*XDL*	XynR	actagtaaacgcttacgata	14	Xylan ABC transporters, endoxylanases, β-xylosidases, and esterase
*aguX-uxuBA-bgaL*	XynR	tttagaaatcgttttctaaa	4	Xylan α-glucuronidase, glucuronate catabolism, and β-galactosidase
*xylA*	XylR	tagtttgtttaataaacaaacta	1	Xylose isomerase
*xynUVW-xylR-xynA*	XylR	tagtttgtttaagaaataaacca	5	XOS transporter, XylR regulator, and endoxylanase

**Fig 2:**
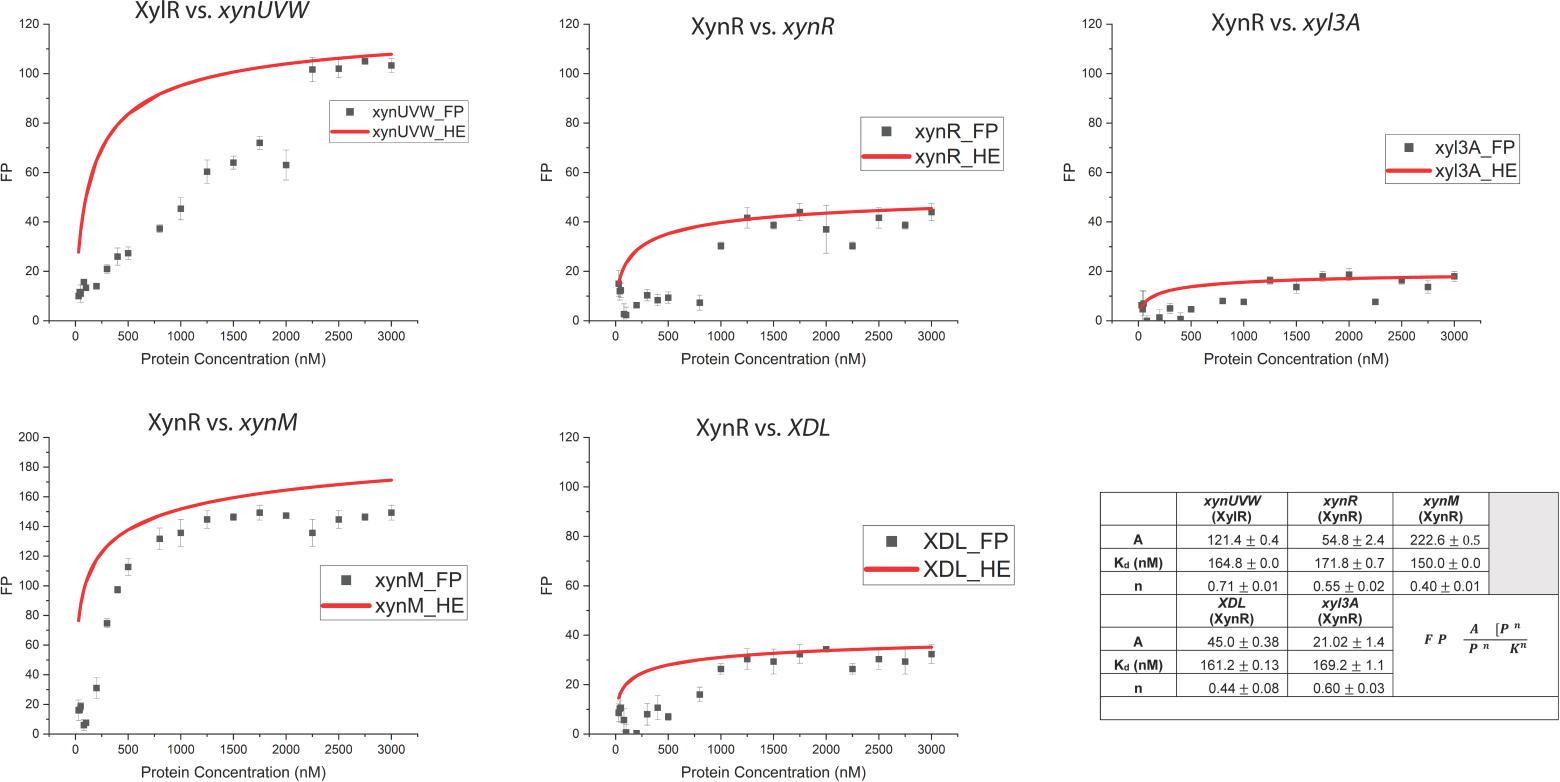
Fluorescence polarization (FP) of XynR and XylR binding to sequence motifs (Table 3). Data were fit to the Hill equation (in red): A = amplitude for ΔFP, [P] = protein concentration (nM), K_d_ = dissociation constant (nM), and *n* = Hill Coefficient.

**Fig 3:**
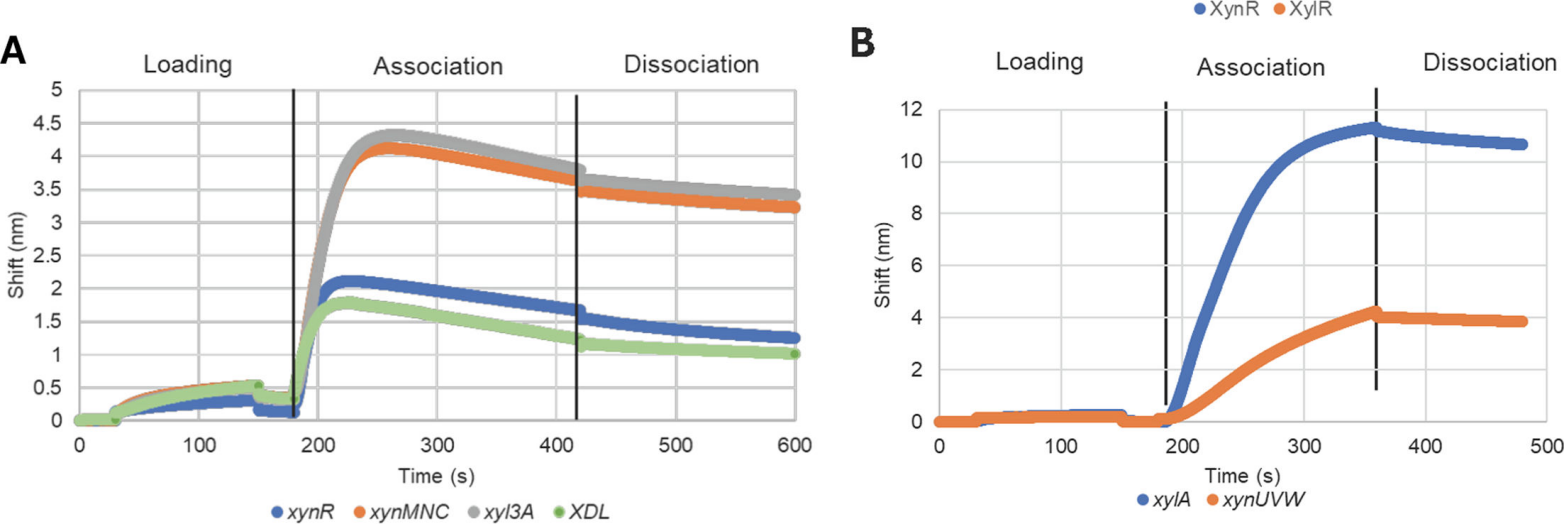
Confirmation of protein-DNA interactions by biolayer interferometry for XynR and XylR. Representative BLI sensograms for XynR against the *xynR, xynMNC, xyl3A, and XDL* operator sites (A) and XylR against the *xylA* (xylose isomerase) and *xynUVW* operator sites (B), with sequences shown in Table 4.

**Fig 4:**
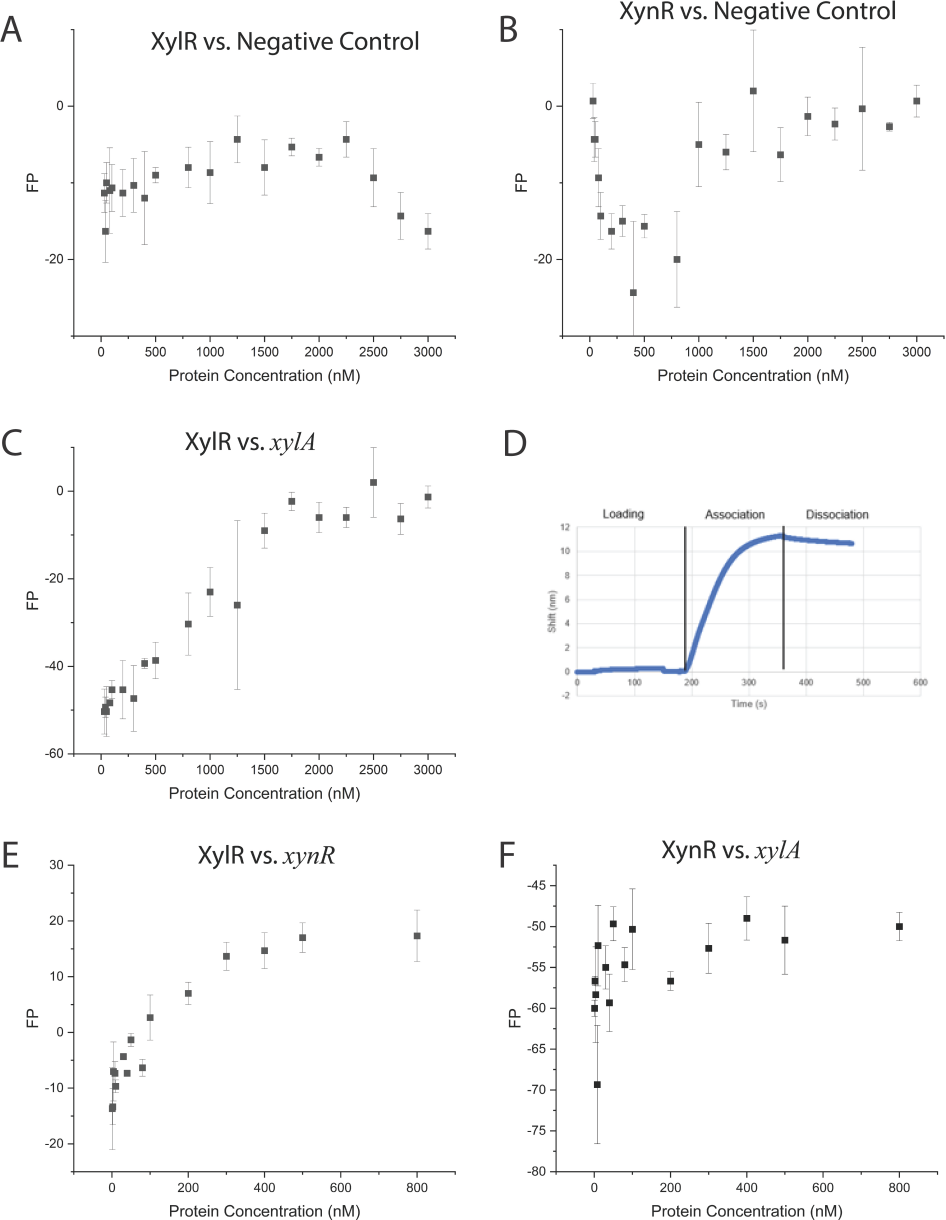
Negative controls for fluorescence polarization (FP) of XylR (**A**), XynR (**B**), and *xylA* showing no indication of binding to XylR (**C**) and XylR binding to *xylA* using BLI (**D**), Fluorescence polarization results for XylR against *xynR,* a XynR regulator (**E**), and XynR against *xylA*, a XylR regulator (**F**). All sequences shown in Tables 3–4.

From FP analysis, a Hill Coefficient model indicated that XylR binds to *xynUVW* with a K_d_ = 165 nM, but not to *xylA*. XynR binds to *xynR* (K_d_ = 172 nM), *xynM* (K_d_ = 150 nM), *XDL* (K_d_ = 161 nM), and *xyl3A* (K_d_ = 169 nM) (Fig. 2). The Hill Coefficient in all cases was less than 1, suggesting negative cooperativity or decreased affinity of other binding sites for the transcription factor. To further evaluate XylR binding to *xylA*, BLI analysis was performed, which did, as expected from bioinformatic analysis, confirm the association (Fig. 3). In the case of inconclusive results for binding between XylR and *xylA* in FP results, the binding was confirmed using BLI (Fig. 1). Negative controls using random DNA sequences and cross-checks between XynR and XylR regulators are shown in Fig. 4.

**Fig 5:**
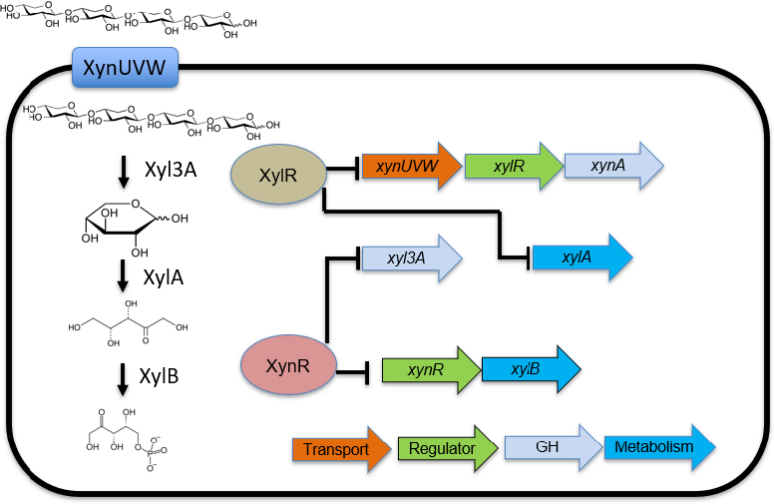
Regulatory model for intersecting XynR and XylR regulons. Transport of linear xylan is performed by XynUVW, catabolism of xylooligosaccharides (XOS) is performed by Xyl3A, and then free xylose is converted to xylulose-5-phosphate by the xylose isomerase-xylulose pathway. XylR represses the expression of *xynUVW* and *xylA,* while XynR controls *xyl3A* and *XylB*. The interconnected regulation on the four-step transport and conversion of XOS highlight the importance of these two transcription factors for xylan metabolism.

These results suggest several features of the regulation of xylan metabolism by *A. bescii* (Fig. 5). XynR co-regulates the expressions of *xyl3A*, *xynM*, *xynR*, and *XDL*, which collectively encode ABC transporters and catabolic enzymes involved in xylan degradation. Previous work showed that Xyl3A is the primary β-xylosidase used by *Caldicellulosiruptorales* species for conversion of XOS into monomeric D-xylose, in coordination with the XDL that encodes several secreted endoxylanases (17). Furthermore, XynR is likely co-operonic with the xylulose kinase and regulates this gene pair. This implies that XynR controls several important hemicellulosic processes and the ATP-dependent step of xylose catabolism. However, XylR, and not XynR, controls the expression of the putative XOS/xylose transporter (*xynUVW*).

### Comparative analysis of XynR and XylR homologs across xylanolytic thermophiles

**Fig 6:**
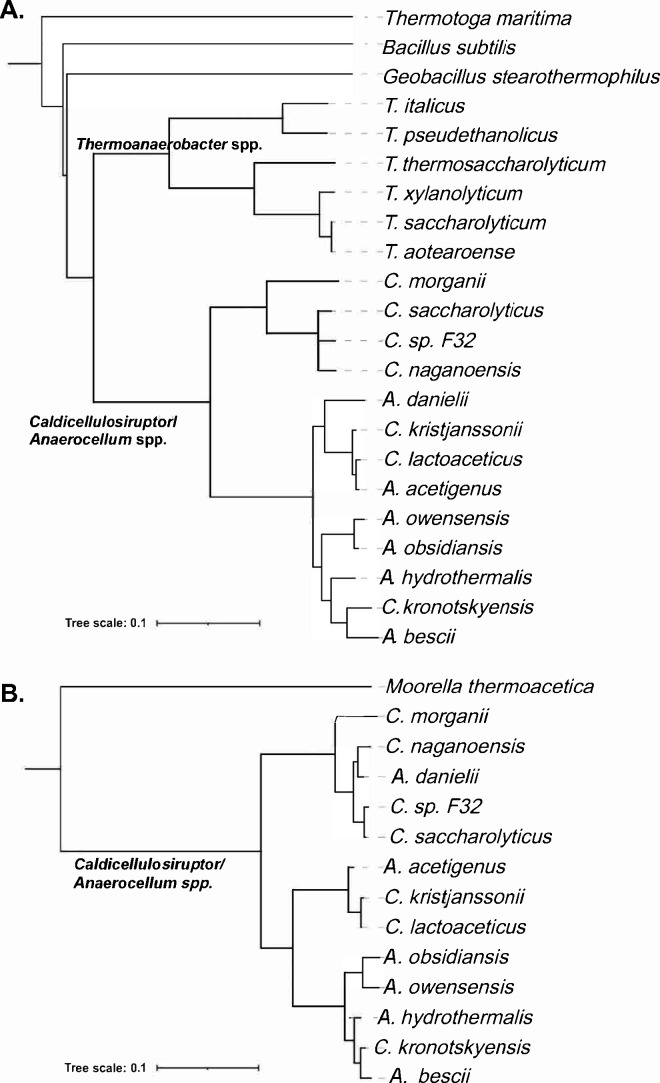
Phylogenetic tree of XylR and XynR protein orthologs in *Caldicellulosiruptorales* and related bacterial species. Phylogenetic trees were constructed in MEGA for XynR (Panel A) and XylR orthologs (Panel B).

Across the *Caldicellulosiruptorales*, orthologs to both XynR and XylR from *A. bescii* are found in all sequenced genomes in this order (Fig. 6). The overall amino acid identity (AAI) of these transcription factors across the genus is greater than 78% for all species for XynR and greater than 70% for XylR. Genomic searches for XylR and XynR binding sites led to the reconstruction of both transcriptional regulons in the other *Caldicellulosiruptorales* genomes. The reconstructed XylR regulon includes both *xylA* and *xynUVW* targets in nine genomes, including *A. bescii and Caldicellulosiruptor saccharolyticus*. Three other genomes corresponding to other species in this order do not have the *xylA* gene in their genomes, and their reconstructed XylR regulons include only orthologs of *xynUVW* genes. The *xylA2* gene (e.g., Csac_1154 in *C. saccharolyticus*), encoding non-orthologous replacement of xylulose isomerase XylA, is not co-regulated via XylR or XynR. The reconstructed XynR regulons include the *xynR-xylB*, *xyl3A*, and *xynMNC* operons in all *Caldicellulosiruptorales* genomes (the regulon core genes), while the *aguX-uxuBA-bgaL* operon is also preceded by a conserved XynR binding site in all genomes except *Caldicellulosiruptor naganoensis* NA10 and *Caldicellulosiruptor* sp. F32 strains that apparently have lost this glucuronide catabolic operon from their genomes. The XDL operon was identified in 11 *Caldicellulosiruptorales* genomes. However, it is preceded by a highly scored XynR binding site in *A. bescii*, *Anaerocellum kronotskyensis*, and *C. saccharolyticus* and by a lowly scored site in *Anaerocellum acetigenus* and *Anaerocellum owensensis*. In six other genomes, the site does not have a XynR operator and is potentially controlled by another local regulator from the LacI family encoded within the XDL locus. Thus, from an evolutionary standpoint, the XDL locus has the most flexible regulatory interactions within species in the Order *Caldicellulosiruptorales*.

Analysis of the genomic context of XylR and XynR orthologs in other bacterial taxa showed several major differences in the corresponding regulons. The closest orthologs to *A. bescii* XylR with amino acid identity ~48% were identified in thermophilic clostridia from the genus *Thermoanaerobacter* (Fig. 6A), where they presumably control the xylose/XOS utilization gene cluster (Fig. 6). Previously characterized xylose-responsive repressors from *Bacillus subtilis* and *Geobacillus stearothermophilus* are each 43% identical to XylR from *A. bescii* and have very similar consensus DNA-binding motifs with *A. bescii* XylR. The XylR repressor in *B. subtilis* controls the xylose catabolic genes *xylAB* and XOS utilization operon *xynP-xynB* (PMID: 1588910), while the *G. stearothermophilus* XylR regulon includes at least five different gene loci encoding xylan degradation enzymes, XOS transporter and catabolic genes, as well as the *xylAB* operon and xylose mutarotase XylM (Fig. 6) (18). Interestingly, transcriptional regulation of xylan degradation genes in *G. stearothermophilus* also involves a two-component regulatory system for XOS ABC transporter (XynDC) and a putative regulator for xylanase (XynX) (18). Another previously characterized xylose-responsive regulator from the ROK family in hyperthermophilic bacterium *Thermotoga maritima* is 33% identical to the *A. bescii* XylR protein and co-regulates the xylose utilization genes, with multiple gene loci encoding xylan degradation enzymes and XOS transporters (Fig. 5) (17).

However, in the case of XynR, the closest homolog outside the *Caldicellulosiruptorales* is in *Moorella thermoacetica*, which has 38% amino acid identity to the *A. bescii* XynR protein and is encoded within the *xylAB-xynR-xylM-xylFGH* gene cluster, preceded by a candidate XynR-binding site. Reconstruction of the *M. thermoacetica* XynR regulon did not identify other potential targets in the genome, suggesting XynR is a local regulator of xylose utilization genes in this organism. Besides *Caldicellulosiruptorales* and *Moorella*, XynR orthologs have not been identified in other taxa of Firmicutes. Additional homologs (< 35% AAI), found in *Halobacillus aidingensis*, *Brevibacillus agri*, and unidentified *Firmicutes* and *Chloroflexi*, are not clustered on the chromosome with xylose/xylan catabolic genes and thus unlikely to control xylose metabolism. Despite the presence of XylR for controlling a small fraction of the xylanolytic genes in *A. bescii*, XynR is the major regulator for xylan and xylose metabolism in *Caldicellulosiruptorales* spp., and this protein appears to have no characterized homologs that perform a similar function.

## DISCUSSION

The role of two xylanolytic transcription factors, XynR and XylR, from *A. bescii* was investigated experimentally to validate previous bioinformatic predictions (11). XynR bound to all four of the tested *in silico* predicted sites, while XylR was found to bind both of the sites tested. As these proteins are conserved across the Order *Caldicellulosiruptorales*, the results here provide additional insights into how *Caldicellulosiruptorales* species regulate their xylanolytic inventories and central xylan metabolism.

**Fig 7:**
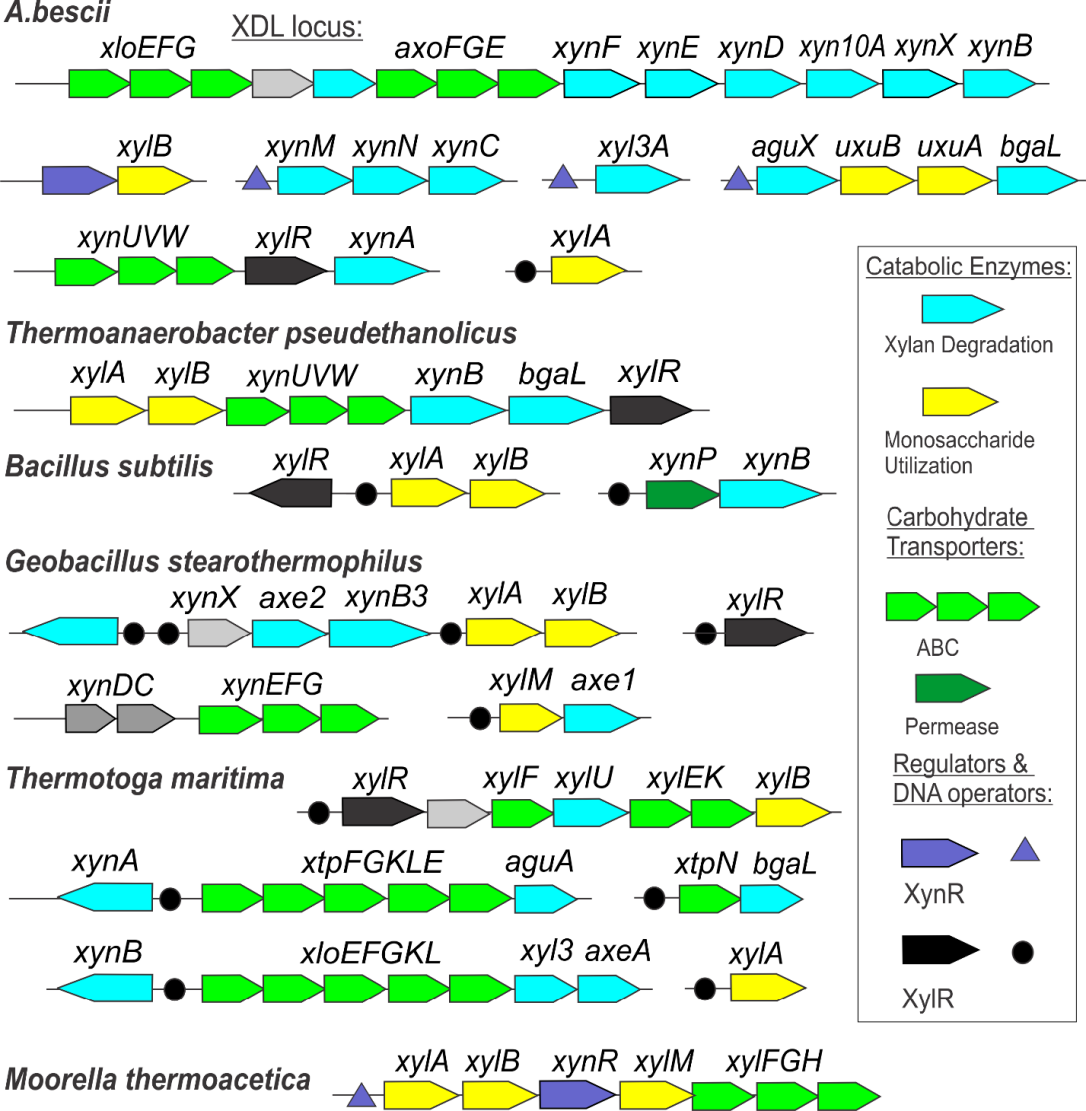
Genomic content of xylose/xylan regulons in *A. bescii* and other gram-positive bacteria.

The regulation of xylan and xylose metabolism has been investigated in several thermophilic bacteria (19, 20) and in a thermophilic archaeon (21). XynR and XylR homologs appear in other thermophilic species; for example, XylR orthologs are present in *Geobacillus stearothermophilus*, a well-characterized xylan degrader (Fig. 7). The *G. stearothermophilus* XylR controls more genes than the *A. bescii* XylR and has more binding sites in the genome (18). Conversely, the closest homolog to *A. bescii* XynR outside of the genus is in *M. thermoacetica*, which belongs to the phylum Firmicutes, that utilizes both gaseous and carbohydrate-derived carbon substrates for growth. Additionally, no homologs of this protein have been characterized, so its role for *A. bescii* as a global repressor may provide insights into how other organisms with this protein may regulate their metabolism. In *Caldicellulosiruptorales* species, the relatively weak repression by XynR suggests that any changes in the available carbohydrates in the environment will allow for rapid adaptation to new growth substrates.

The implications for two regulatory strategies for xylan utilization highlight a larger theme for *Caldicellulosiruptorales* species. While *A. bescii* does not exhibit carbon catabolite repression nor does have a homolog to CcpA, it instead encodes numerous transcription factors to detect the available sugars and activate the appropriate machinery to process those sugars. The parallel regulatory strategies of XynR and XylR (Fig. 5) emphasize simultaneous control of ATP consumption by *Caldicellulosiruptorales* species. XylR controls the primary transporter for xylooligosaccharides and one of the steps in xylan catabolism. Furthermore, XylR regulates the expression of a secreted xylanase that is highly active on linear xylan, providing a similar strategy to *G. stearothermophilus* XylR. The released xylooligosaccharides then likely activate the XynR regulon, which regulates the expression of additional xylanases that are more active on heterogeneous parts of lignocellulose and all of the β-xylosidases encoded in *A. bescii*. XynR additionally controls xylulose kinase, which links xylose utilization to central carbon metabolism. The regulation on xylose isomerase by XylR acts as a feedback loop to control both regulons. Unfortunately, effectors were unable to be experimentally determined under the conditions tested in this work due to the need to run binding assays at temperatures far below the optimum growth temperature for *Anaerocellum bescii*.

Overall, the validation of TFBS and measurement of affinities for two global transcription factors provides insights into how *Caldicellulosiruptorales* species regulate xylan utilization. This insight should prove useful for further efforts to enhance the development of *A. bescii* into a metabolic engineering platform.

## MATERIALS AND METHODS

### Plasmid construction, maintenance, and protein expression

All genes of interest and the pET28a plasmid backbone were PCR-amplified using Q5 polymerase (New England Biolabs, Ipswich, MA) with suitable overhangs to perform Gibson assembly (Table 1). *A. bescii* genomic DNA was isolated, as previously described (9). Gibson assembly was performed using the NEBuilder HiFi DNA Assembly Mastermix. For both XynR (UniProt Accession Number B9MPD1) and XylR (UniProt Accession Number Q44406), the endogenous stop codon was removed to add a C-terminal 6 x His purification tag. All plasmids were transformed into *E. coli* DH5α, verified by Sanger sequencing (Azenta Life Sciences, Morrisville, NC), and maintained as glycerol stocks in Luria Bertani (LB) medium +15% glycerol +50 µg/mL kanamycin at −80°C. Plasmids were then transformed into *E. coli* Rosetta pLysS with strains maintained in LB medium +15% glycerol +50 µg/mL kanamycin and 34 µg/mL chloramphenicol. Protein expression was induced in a modified 2 x YT medium for induction containing 16 g/L tryptone, 10 g/L yeast extract, 5 g/L NaCl, 0.5 g/L glucose, 0.4% (vol/vol) glycerol, and 2 g/L lactose, plus the appropriate antibiotics at 37°C for 24 h (22). Cells were harvested by centrifugation at 6,000 × *g* for 10 min prior to lysis and purification.

### Protein purification

*E. coli* cell pellets were resuspended with 3 mL/g wet weight cells in 20 mM Tris-HCl, 300 mM sodium chloride, pH 7.6. Resuspended cells were lysed using three passes in a French press (Sim-Aminco) at ~13,000 psig. Because all proteins were expected to be thermostable, lysates were heat-treated at 65°C for 20 min, clarified by centrifugation at 20,000 × *g* for 60 min, and sterile-filtered through a 0.22 µm filter prior to purification.

His-tagged proteins were purified by immobilized metal affinity chromatography (IMAC) using a 500 mM imidazole gradient for elution on a 5 mL HisTrap HP column (Cytiva). All chromatographic procedures were performed on a Biologic DuoFlow FPLC (Bio-Rad) for BLI and NGC Quest (Bio-Rad) for FP. Fraction purity was evaluated by SDS-PAGE by incubating 10 µL of the sample in an equal amount of 2 x Laemmli buffer (Bio-Rad) at 99°C for 20 min. Denatured proteins were resolved on a 4-20% TGX Mini-Protean stain-free gel (BioRad). Protein fractions containing the correct protein size were pooled and concentrated through Vivaspin 20 filters with a 10 kDa cutoff. Proteins were then buffer-exchanged into 20 mM Tris-HCl, 100 mM sodium chloride, 0.02% Tween 20, and pH 7.4 (biolayer interferometry (BLI) buffer herein, and the same buffer composition was used for all FP experiments). The protein concentration was determined using a Qubit 4 fluorometer (Thermo Fisher) and the Pierce BCA assay kit (ThermoFisher) according to the manufacturer’s instructions with bovine serum albumin as the protein standard.

### Operator probe design and synthesis

Predicted operator sites for XylR and XynR were previously reported by Rodionov et al. (14). The predicted operator site was centered in the oligonucleotide sequence, with the 14 bp of the genomic sequence flanking the operator site on both the 5′ and 3′ ends, to generate probes between 44 bp and 46 bp. Complementary oligonucleotides were ordered from Integrated DNA Technologies (Coralville, IA), with one of the nucleotides having a 5′ biotin end (Table 2). Oligonucleotides were resuspended in BLI buffer to produce 100 µM stocks. Equimolar amounts of each nucleotide (1 µM final concentration) were diluted in BLI buffer, mixed, heated to 95°C for 2 min, and then cooled at 0.5 °C/min to room temperature. Hybridized probes were diluted to 100 nM for use in biolayer interferometry experiments.

**TABLE 2:** Primers used for plasmid construction^*a*^

Primer ID	Purpose	Sequence
JRC 807	pET28a Backbone R	GCCCATGGTATATCTCCTTCTTAAAG
JRC 808	pET28a Backbone F	ACTCGAGCACCACCACC
JRC 833	pET28a-XynR F	ctttaagaaggagatatacc**atg**ggcCCGTCGATTGAGGATGTTGC
JRC 834	pET28a-XynR R	ggtggtggtggtgctcgagTGCAGATTGTCTGATTATAAGCTGAGTG
JRC 835	pET28a-XylR F	ctttaagaaggagatatacc**atg**ggcGGTAACCACACGCTACTAAAG
JRC 836	pET28a-XylR R	ggtggtggtggtgctcgagTGCATATTCTGGAAAAGAAAGC

^*a*^ Lowercase letters represent the Gibson overhang region on pET28; the start codon is underlined and shown in boldface.

### Biolayer interferometry (BLI)

BLI experiments were performed on a BLItz instrument (ForteBio) using streptavidin SA biosensors (Sartorius). Sensors were hydrated for 10 min in BLI buffer, and the BLItz was allowed to warm up for 20 min prior to use. Initial tests of protein binding with 500 nM DNA immobilization and 180 sec association steps produced large shifts (Δλ between 7 and 10 nm) and often did not reach equilibrium. Therefore, 100 nM DNA during the immobilization was used for all subsequent kinetic assays, which is consistent with other BLI investigations (9, 10, 20). Association times ranged from 180 to 240 sec in an effort to balance achieving equilibrium during the trial against having dissociation during the immobilization step. Dissociation steps were performed between 120 and 180 sec to ensure that the final shift was less than 90% of the shift observed at the initiation of dissociation. Separate drop holders were used for DNA and protein in all trials, with 4 µL being loaded for the immobilization and association steps. All baseline steps and the dissociation step were performed in 250 µL of BLI buffer. Measurements were performed in duplicate, biosensors were discarded after each measurement, and drop holders were cleaned between samples with 4 µL of 0.5 M HCl, rinsed twice with deionized water, and dried with precision wipes (Kimberly Clark).

### BLI data analysis

Data analysis was performed in the BLItz software (v1.0.8) to determine the equilibrium shifts.

### Fluorescence polarization (FP)

All oligonucleotides (Table 3) were designed similar to BLI according to the sequences in Table 4 and were obtained from Integrated DNA Technologies (Coralville, IA), with one of the nucleotides having a 5’ 6-carboxyfluorescein (6-FAM) end. Probe hybridization was also carried out similar to the BLI probes in the same buffer. Survey experiments were carried out with varying protein and probe concentrations. For the studied protein concentration range (between 30 nM and 3 µM), a probe concentration of 5 nM led to the best signal strength and was used in all experiments. 2X aliquots of proteins and probes at each concentration were prepared, and 25 µL of each was mixed in Corning 384 Flat Bottom Black Polystyrene plates in triplicates at room temperature (22°C). All FP measurements were carried out using an Infinite 200Pro plate reader (Tecan Life Sciences, NC, USA), with excitation and emission wavelengths of 485 nm and 535 nm, respectively.

**TABLE 3:** Oligonucleotides used for fluorescence polarization^*a*^

Operator site	Nucleotide ID	Sequence (5'–3')
XynR: XDL (Athe_0174)	XDL 6FAM F	(6FAM) attgACTAGTAAACGCTTACGATAtatt
	XDL Unlabeled R	aataTATCGTAAGCGTTTACTAGTcaat
XynR: *xyl3A* (Athe_2354)	Xyl3A 6FAM F	(6FAM) tgagGTTAGAAAGCGGTTTCAAGCaatt
	Xyl3A Unlabeled R	aattGCTTGAAACCGCTTTCTAACctca
XynR: *xynR*-xylB	XynR 6FAM F	(6FAM) taatAATCGAAATCGCTTACAATTcctg
	XynR Unlabeled R	caggAATTGTAAGCGATTTCGATTatta
XynR: *xynMNC* (Athe2722)	XynMNC 6FAM F	(6FAM) atttTCATGAAATCGCTTTCCTATctaa
	XynMNC Unlabeled R	ttagATAGGAAAGCGATTTCATGAAaata
XylR: *xylA* (Athe_0603)	XI 6FAM F	(6FAM) attatagatATTAGTTTGTTTAATAAACAAACTAAGtacacgtact
	XI Unlabeled R	agtacgtgtaCTTAGTTTGTTTATTAAACAAACTAATatctataat
XylR: *xynUVW* (Athe_0614)	XynUVW 6FAM F	(6FAM) taaatatataaataGTTTGTTTAAGAAATAAACCAAAaggagtgatt
	XynUVW Unlabeled R	aatcactcctTTTGGTTTATTTCTTAAACAAACtatttatatatttA
Negative control	NC_6FAM_F	(6FAM)AGGATCCCTGCGAAGAGGGAGGCTATTG
	NC_Unlabeled_R	CAATAGCCTCCCTCTTCGCAGGGATCCT

^*a*^ Predicted operator sites are shown in uppercase letters.

**TABLE 4:** Oligonucleotides used for biolayer interferometry^*a*^

Operator site	Nucleotide ID	Sequence (5'–3')
XynR: XDL (Athe_0174)	XDL Biotin F	(Biotin) caaaaaatattgACTAGTAAACGCTTACGATAtattatataaac
	XDL Unlabeled R	gtttatataataTATCGTAAGCGTTTACTAGTcaatattttttg
XynR: *xyl3A* (Athe_2354)	Xyl3A Biotin F	(Biotin) ctttgtgctgagGTTAGAAAGCGGTTTCAAGCaatttaaaacaa
	Xyl3A Unlabeled R	ttgttttaaattGCTTGAAACCGCTTTCTAACctcagcacaaag
XynR: *xynR*-xylB	XynR Biotin F	(Biotin) ataacaaagaatAATCGAAATCGCTTACAATTcctgtatttgat
	XynR Unlabeled R	atcaaatacaggAATTGTAAGCGATTTCGATTattctttgttat
XynR: *xynMNC* (Athe2722)	XynMNC Biotin F	(Biotin) atatttttttatttTCATGAAATCGCTTTCCTATctaaaatattaa
	XynMNC Unlabeled R	ttaatattttagATAGGAAAGCGATTTCATGAAaataaaaaaatat
XylR: *xylA* (Athe_0603)	XI Biotin F	(Biotin) attatagatATTAGTTTGTTTAATAAACAAACTAAGtacacgtact
	XI Unlabeled R	agtacgtgtaCTTAGTTTGTTTATTAAACAAACTAATatctataat
XylR: *xynUVW* (Athe_0614)	XynUVW Biotin F	(Biotin) gaaatatataaataGTTTGTTTAAGAAATAAACCAAAaggagtgatt
	XynUVW Unlabeled R	aatcactcctTTTGGTTTATTTCTTAAACAAACtatttatatatttc

^*a*^ Predicted operator sites are shown in uppercase letters.

### FP data analysis

The following iteration of the Hill equation was used to fit the FP results in Microsoft Excel, using Solver: $FP=\frac{A*{\left\lfloor P\right\rfloor }^{n}}{{\left\lfloor P\right\rfloor }^{n}+K_{d}^{n}}$ , where FP is the fluorescence polarization signal value, A is the amplitude of change in FP, [P] is the protein concentration (nM), and K_d_ is the dissociation constant (nM).
